# Spread of infection and treatment interruption among Japanese workers during the COVID-19 pandemic: A cross-sectional study

**DOI:** 10.3389/fpubh.2022.921966

**Published:** 2022-07-29

**Authors:** Jun Akashi, Ayako Hino, Seiichiro Tateishi, Tomohisa Nagata, Mayumi Tsuji, Akira Ogami, Shinya Matsuda, Masaharu Kataoka, Yoshihisa Fujino

**Affiliations:** ^1^Second Department of Internal Medicine, School of Medicine, University of Occupational and Environmental Health, Kitakyushu, Japan; ^2^Department of Mental Health, Institute of Industrial Ecological Sciences, University of Occupational and Environmental Health, Kitakyushu, Japan; ^3^Department of Occupational Medicine, School of Medicine, University of Occupational and Environmental Health, Kitakyushu, Japan; ^4^Department of Occupational Health Practice and Management, Institute of Industrial Ecological Sciences, University of Occupational and Environmental Health, Kitakyushu, Japan; ^5^Department of Environmental Health, School of Medicine, University of Occupational and Environmental Health, Kitakyushu, Japan; ^6^Department of Work Systems and Health, Institute of Industrial Ecological Sciences, University of Occupational and Environmental Health, Kitakyushu, Japan; ^7^Department of Preventive Medicine and Community Health, School of Medicine, University of Occupational and Environmental Health, Kitakyushu, Japan; ^8^Department of Environmental Epidemiology, Institute of Industrial Ecological Sciences, University of Occupational and Environmental Health, Kitakyushu, Japan

**Keywords:** COVID-19, patient acceptance of health care, treatment refusal, regional medical programs, Japan

## Abstract

**Background:**

The COVID-19 pandemic has resulted in treatment interruption for chronic diseases. The scale of COVID-19 in Japan has varied greatly in terms of the scale of infection and the speed of spread depending on the region. This study aimed to examine the relationship between local infection level and treatment interruption among Japanese workers.

**Methods:**

Cross-sectional internet survey was conducted from December 22 to 26, 2020. Of 33,302 participants, 9,510 (5,392 males and 4,118 females) who responded that they required regular treatment were included in the analysis. The infection level in each participant's prefecture of residence was assessed based on the incidence rate (per 1,000 population) and the number of people infected. Age-sex and multivariate adjusted odds ratios (ORs) of regional infection levels associated with treatment interruption were estimated by multilevel logistic models, nested by prefecture of residence. The multivariate model was adjusted for sex, age, marital status, equivalent household income, educational level, occupation, self-rated health status and anxiety.

**Results:**

The ORs of treatment interruption for the lowest and highest levels of infection in the region were 1.32 [95 % confidence interval (CI) were 1.09–1.59] for the overall morbidity rate (per 1,000) and 1.34 (95 % CI 1.10–1.63) for the overall number of people infected. Higher local infection levels were linked to a greater number of workers experiencing treatment interruption.

**Conclusions:**

Higher local infection levels were linked to more workers experiencing treatment interruption. Our results suggest that apart from individual characteristics such as socioeconomic and health status, treatment interruption during the pandemic is also subject to contextual effects related to regional infection levels. Preventing community spread of COVID-19 may thus protect individuals from indirect effects of the pandemic, such as treatment interruption.

## Introduction

COVID-19, first identified at the end of 2019, is continuing to rage around the world (1–4). Having experienced four waves of the disease through June 2021 there is an urgent need in Japan to understand the working and social environment and the health status of workers affected by the COVID-19 pandemic. In addition to direct effects of severe pneumonia and acute respiratory failure, COVID-19 has also had indirect health effects. COVID-19-related treatment interruption, particularly in patients with chronic diseases, is an emerging issue in several countries (5, 6), including Japan (7). Studies have reported a significant decrease in the number of prescriptions during the pandemic compared to before, and that 40 % of patients requiring regular visits have been seen less frequently (8, 9).

Treatment interruption for diseases other than COVID-19, which should normally be continued, can cause serious health care problems in several ways. First, it can exacerbate the medical condition of patients with chronic diseases that require regular management. Second, few opportunities for regular physical examinations may lead to undiagnosed complications and delayed treatment. Further, such medical problems, which could have been avoided by continued treatment, may cause further strain on future health care resources (10). Studies performed during the COVID-19 pandemic have reported that treatment interruption among patients with chronic diseases is associated with a variety of factors, including fear of becoming infected when seeing the doctor (6, 11), scheduling changes by hospitals (12, 13), and shortage of medical resources (6). These factors presumably have differing degrees of impact depending on the level of infection in the region, such as incidence rate and cumulative COVID-19 cases. In addition, patients with unstable socioeconomic status are more likely to discontinue treatment (7, 14, 15). Areas with higher prevalence of COVID-19 may be more affected by the loss of job security and other factors that affect individuals with unstable socioeconomic status.

In Japan, the spread of COVID-19 has varied widely by region in terms of the scale of infection and the speed of spread (16, 17). We hypothesize that differences in regional infection rates will affect treatment interruption in each region. The level of infection in a community, such as incidence rate and the number of people with COVID-19, may directly or indirectly affect fear of visiting medical institutions, anxiety about going out, and financial difficulties, which may cause treatment interruption. For example, the number of people with COVID-19 is reported daily by region. Such information will arouse some degree of anxiety and fear in people living in regions with high levels of infection about the safety of the area and the disease. Tokyo, which has recorded the greatest number of infections in Japan, saw a significant drop in prescriptions through May 2020 (8). Given that pandemics are known to overwhelm medical resources (18), Japan's lack of capacity to conduct COVID-19 tests in areas with high levels of infection and limited hospital beds has exposed the limits of the country's medical resources (19).

However, the relationship between regional COVID-19 infection level and treatment interruption remains to be elucidated. Japan provides an ideal opportunity to test our hypotheses due to the country's large regional variation in COVID-19 infection levels. Therefore, this study investigated the relationship between both local viral infection levels measured and treatment interruptions described, in Japan.

## Materials and methods

### Study design and subjects

Cross-sectional internet survey was conducted from December 22nd to 26th, 2020, the period corresponding to Japan's third wave of infection, as a part of the Collaborative Online Research on the Novel-coronavirus and Work (CORoNaWork) Project (20). The target population was formed by workers aged 20–65 years at the time of this survey. Data were obtained from participants who indicated that they were employed at the time of the survey, with participants selected based prefecture of residence, job type, and sex. A detailed description of the protocol of this survey is provided elsewhere (20). Of the 33,302 participants in the survey, 6,266 were excluded for providing fraudulent responses. Of the 27,036 remaining participants, data from 9,510 (5,392 males and 4,118 females) who described themselves as needing regular treatment or hospital visits were analyzed.

This study was approved by the Ethics Committee of the University of Occupational and Environmental Health, Japan (Reference Nos. R2-079 and R3-006) and performed in accordance with relevant guidelines and regulations. Participants provided informed consent by completing a form on the survey website.

### Treatment status

This study used a single-item question to assess participants' treatment status: “Do you have a condition that requires regular hospital visits or treatment?” Participants check from “I do not have such a condition,” “I am continuing with hospital visits and treatment as scheduled,” and “I am not able to continue with hospital visits and treatment as scheduled.”

### Infection level indices

The infection level in each participant's prefecture of residence was assessed based on the incidence rate for the entire period from January 2020, when the first case was identified in Japan, to December 16th, 2020; the number of people infected for the entire period; the incidence rate in the month before the survey (per 1,000 population); and the number of people infected over that same month. These values were calculated using publicly available data from the Ministry of Health, Labor and Welfare (16).

### Socioeconomic status, health status, and anxiety

Socioeconomic status, health status, and anxiety were assessed through questionnaires in the Internet survey. Socioeconomic factors were age, sex, marital status (married, unmarried, bereaved/divorced), occupation (mainly desk work, mainly interpersonal communication, mainly labor), education (graduated from junior high school, high school, vocational school/college, university, graduate school), and equivalent income [household income divided by the square root of household size; 500,000–2,650,000, 2,650,000–4,500,000, >4,500,000 Japanese Yen (JPY)]. Health and psychological factors were assessed by self-report (very good, neither good nor bad, not good). Anxiety about contracting COVID-19 was assessed using the following question: “Do you feel anxious about being infected with COVID-19?” Participants chose from “yes” or “no”.

### Statistical analysis

We estimated age-sex- and multivariate-adjusted odds ratios (ORs) of treatment interruption associated with regional infection level by nesting multilevel logistic models in prefecture of residence. This study used four indices of regional infection level: incidence rate for the entire period (per 1,000 population), number of people infected for the entire period, incidence rate in 1 month (per 1,000 population), and number of people infected in 1 month. For analysis, these indices were divided into quartiles and used as area-level variables. In the multivariate model, sex, age, marital status, job type, equivalent household income, education, self-rated health, and anxiety were adjusted. *p* < 0.05 indicated statistical significance. All analyses were conducted using Stata (Stata Statistical Software: Release 16; StataCorp LLC, TX, USA).

## Results

The participants' characteristics together with residential area according to the number of people infected for the entire period are summarized in Table 1. This study stratified the 9,510 participants in need of regular treatment into four groups according to the regional infection level. Socioeconomic factors including sex, age, marital status, household income, education, and occupation in each group, as well as self-assessment of health status and anxiety related to COVID-19 infection are shown. The group with the highest number of people infected tended to have higher annual equivalent household income, and a higher percentage were in vocational school/college, university, and graduate school.

**Table 1:** Basic characteristics of the study subjects.

	**Residential area according to the number of people infected for the entire period**			
	**74–492**	**507–1,496**	**1,673–11,982**	**12,381–52,382**
Number of subjects	2,130	2,579	2,422	2,379
Age, median [interquartile range (IQR)]	51 (42, 57)	51 (43, 57)	52 (44, 58)	53 (46, 58)
Sex, male	1,181 (55.4 %)	1,440 (55.8 %)	1,403 (57.9 %)	1,368 (57.5 %)
Marital status, married	1,252 (58.8%)	1,496 (58.0 %)	1,370 (56.6 %)	1,284 (54.0 %)
Annual equivalent household income (JPY)				
500,000–2,650,000	773 (36.3 %)	875 (33.9 %)	816 (33.7 %)	717 (30.1 %)
2,650,000–4,500,000	690 (32.4 %)	824 (32.0 %)	733 (30.3 %)	648 (27.2 %)
>4,500,000	667 (31.3 %)	880 (34.1 %)	873 (36.0 %)	1,014 (42.6 %)
Education				
Junior high school	26 (1.2 %)	32 (1.2 %)	30 (1.2 %)	36 (1.5 %)
High school	703 (33.0 %)	750 (29.1 %)	619 (25.6 %)	500 (21.0 %)
Vocational school/college, university, graduate school	1,401 (65.8 %)	1,797 (69.7 %)	1,773 (73.2 %)	1,843 (77.5 %)
Occupation				
Mainly desk work	1,144 (53.7 %)	1,293 (50.1 %)	1,222 (50.5 %)	1,264 (53.1 %)
Mainly interpersonal communication	480 (22.5 %)	590 (22.9 %)	622 (25.7 %)	614 (25.8 %)
Mainly labor	506 (23.8 %)	696 (27.0 %)	578 (23.9 %)	501 (21.1 %)
Self-rated health				
Very good	742 (34.8 %)	895 (34.7 %)	895 (37.0 %)	885 (37.2 %)
Neither good nor bad	919 (43.1 %)	1,104 (42.8 %)	991 (40.9 %)	986 (41.4 %)
Not good	469 (22.0 %)	580 (22.5 %)	536 (22.1 %)	508 (21.4 %)
Do you feel anxious about being infected with COVID-19?				
Yes	1,684 (79.1 %)	2,083 (80.8 %)	1,904 (78.6 %)	1,850 (77.8 %)
The incidence rate for the entire period (per 1,000 of the population), median (IQR)	0.28 (0.22, 0.34)	0.55 (0.51, 0.59)	1.26 (0.79, 1.51)	3.12 (1.91, 3.76)
The number of people infected for the entire period, median (IQR)	379 (330, 445)	1,053 (671, 1,124)	2,455 (2,168, 8,438)	27,500 (14,427, 52,382)
The incidence rate in the month before the survey (per 1,000 of the population), median (IQR)	0.09 (0.058, 0.14)	0.23 (0.16, 0.32)	0.47 (0.33, 0.59)	1.06 (0.74, 1.06)
The number of people infected in the month before the survey, median (IQR)	124 (39, 171)	440 (282, 501)	1,705 (916, 2,936)	9,851 (5,596, 14,690)
The number of people who had interrupted treatment	220 (10.3 %)	285 (11.1 %)	269 (11.1 %)	285 (12.0 %)

The prefectures and participants belonging to each infection level indices are shown in Table 2 and the association between the regional infection level and treatment interruption is summarized in Table 3. According to multivariate analysis, the ORs of treatment interruption for the lowest and highest levels were 1.32 (95 % CI: 1.09–1.59; *p* = 0.003) for the overall incidence rate (per 1,000 population), 1.34 (95% CI: 1.10–1.63; *p* = 0.002) for the overall number of people infected, 1.28 (95 % CI: 1.06–1.54; *p* = 0.013) for the monthly incidence rate (per 1,000 population), and 1.38 (95 % CI: 1.14–1.67; *p* = 0.001) for the number of people infected per month. For each index of infection level, a higher infection level was linked to more workers experiencing treatment interruption for chronic diseases in Japan. The results remained unchanged after adjusting for age and sex.

**Table 2:** The prefectures and participants belonging to each infection level indices.

	**Prefecture**	**Total** **(*****n*** = **9,510)**
**The incidence rate for the entire period (per 1,000)**		
0.10–0.44	Aomori, Akita, Ehime, Fukui, Fukushima, Kagawa, Iwate, Nagasaki, Niigata, Shimane, Tokushima, Tottori, Yamaguchi, Yamagata	2,185
0.49–0.68	Kagoshima, Mie, Miyazaki, Nagano, Oita, Okayama, Saga, Shiga, Shizuoka, Tochigi, Toyama, Wakayama, Yamanashi	2,539
0.76–1.63	Chiba, Fukuoka, Gunma, Gifu, Kochi, Kumamoto, Kyoto, Hiroshima, Hyogo, Ibaraki, Ishikawa, Miyagi, Nara, Saitama	2,369
1.89–3.76	Aichi, Hokkaido, Kanagawa, Okinawa, Osaka, Tokyo	2,417
**The number of people infected for the entire period**		
74–492	Akita, Aomori, Ehime, Fukui, Iwate, Kagawa, Nagasaki, Niigata, Saga, Shimane, Tokushima, Tottori, Yamaguchi, Yamagata, Yamanashi	2,130
507–1,496	Fukushima, Ishikawa, Kagoshima, Kochi, Kumamoto, Mie, Miyazaki, Nagano, Oita, Okayama, Shiga, Tochigi, Toyama, Wakayama	2,579
1,673–11,982	Chiba, Fukuoka, Gifu, Gunma, Kyoto, Hiroshima, Hyogo, Ibaraki, Miyagi, Nara, Okinawa, Saitama, Shizuoka	2,422
12,381–52,382	Aichi, Hokkaido, Kanagawa, Osaka, Tokyo	2,379
**The incidence rate in the month before the survey (per 1000)**		
0.018–0.15	Akita, Ehime, Fukui, Fukushima, Ishikawa, Kagawa, Nagasaki, Niigata, Saga, Shimane, Tokushima, Tottori, Toyama, Yamaguchi	2,360
0.16–0.32	Ibaraki, Iwate, Kagoshima, Kumamoto, Mie, Miyagi, Miyazaki, Nagano, Oita, Shiga, Tochigi, Wakayama, Yamagata, Yamanashi	2,269
0.33–0.61	Chiba, Fukuoka, Gifu, Gunma, Hiroshima, Kochi, Kyoto, Nara, Okayama, Saitama, Shizuoka	2,209
0.66–1.12	Aichi, Hokkaido, Kanagawa, Okinawa, Osaka, Hyogo, Tokyo	2,672
**The number of people infected in the month before the survey**		
13–203	Akita, Aomori, Ehime, Fukui, Kagawa, Ishikawa, Nagasaki, Niigata, Saga, Shimane, Tokushima, Tottori, Toyama, Wakayama, Yamaguchi, Yamanashi	2,255
204–626	Fukushima, Kagoshima, Kochi, Kumamoto, Mie, Nagano, Okayama, Oita, Iwate, Miyazaki, Shiga, Tochigi, Yamagata	2,454
704–4,373	Chiba, Fukuoka, Gifu, Gunma, Hiroshima, Hyogo, Ibaraki, Kyoto, Miyagi, Nara, Okinawa, Saitama, Shizuoka	2,422
5,218–14,690	Aichi, Hokkaido, Kanagawa, Osaka, Tokyo	2,379

**Table 3:** Association between regional COVID-19 infection level and treatment interruption.

	**Age-sex adjusted**				Multivariate*			
	**OR**	**95 %, CI**		***P*** **-value**	**OR**	**95 %, CI**		***P*** **-value**
**The incidence rate for the entire period (per 1,000)**								
0.10–0.44	reference				reference			
0.49–0.68	1.00	0.83	1.21	0.999	1.00	0.83	1.21	0.993
0.76–1.63	1.06	0.88	1.28	0.555	1.07	0.88	1.30	0.505
1.89–3.76	1.25	1.04	1.50	0.019	1.32	1.09	1.59	0.005
				0.013^†^				0.003^†^
**The number of people infected for the entire period**								
74–492	reference				reference			
507–1,496	1.07	0.89	1.29	0.473	1.06	0.87	1.29	0.545
1,673–11,982	1.14	0.94	1.38	0.180	1.15	0.94	1.40	0.168
12,381–52,382	1.28	1.06	1.55	0.011	1.34	1.10	1.63	0.003
				0.008^†^				0.002^†^
**The incidence rate in the month before the survey (per 1,000)**								
0.018–0.15	reference				reference			
0.16–0.32	1.07	0.89	1.29	0.487	1.07	0.89	1.30	0.470
0.33–0.61	1.05	0.87	1.27	0.641	1.06	0.87	1.29	0.576
0.66–1.12	1.21	1.02	1.45	0.032	1.28	1.06	1.54	0.009
				0.044^†^				0.013^†^
**The number of people infected in the month before the survey**								
13–203	reference				reference			
204–626	1.11	0.92	1.34	0.284	1.12	0.93	1.36	0.241
704–4,373	1.16	0.96	1.40	0.127	1.18	0.97	1.43	0.093
5,218–14,690	1.30	1.08	1.57	0.006	1.38	1.14	1.67	0.001
				0.006^†^				0.001^†^

^*^The multivariate model was adjusted for age, sex, marital status (married, unmarried, bereaved/divorced), equivalent household income (500,000–2,650,000, 2,650,000–4,500,000, >4,500,000 JPY), educational level (graduated from junior high school, high school, vocational school/college, university, graduate school), occupation (mainly desk work, mainly interpersonal communication, mainly labor), self-rated health (very good, neither good nor bad, not good) and anxiety about infection.

^†^p for trend.

## Discussion

This study showed that regional indices of the scale of infections related to COVID-19 in Japan were correlated with more workers with diseases requiring regular hospital visits and treatment experiencing treatment interruption. To our knowledge, this is the first report showing that community infection levels are associated with treatment interruption.

The COVID-19 pandemic is affecting individuals' socioeconomic status, which is determined by factors such as employment instability. Higher levels of infection have greater socioeconomic impact than lower levels, for example, being more likely to lead to an increase in unemployment and workers in precarious employment situations, which may be a factor affecting treatment interruption (7). Our findings are consistent with those of a previous study showing that such individual factors influence treatment interruption. It is important to emphasize that the association between infection level and treatment interruption remained after adjusting for individual factors such as socioeconomic and health status. These results suggest that apart from individual characteristics, treatment interruptions during the COVID-19 pandemic were also subject to contextual effects related to regional infection levels. For example, rescheduling by medical institutions and health care providers is expected to occur in areas with higher infection levels than in areas with lower levels. Although more research is required to clarify the mechanisms by which regional infection levels lead to treatment interruptions, our study demonstrates that local spread of COVID-19 infection may affect the behavioral characteristics of workers living in the area. These findings suggest that, in addition to an individual patient approach, a population strategy is also needed to prevent the spread of infection and to avoid treatment interruption for manageable diseases.

In this study, both the number of infected people and the infection rate by region were associated with treatment interruption. This suggests that it would be informative to report the incidence rate based on the infection status in each region, which reflects the population of that region. However, Japanese news reports tend to emphasize the number of people infected rather than the infection rate by region, the latter of which may contribute to changing the behavior of more people. A previous study reported that Japanese people have greater trust in local information (21), suggesting that reporting the number of infections by region will have a strong influence on individual's behavioral changes and risk perception.

Increased treatment interruption in areas with high levels of infection may cause further strain on future health care resources. Delaying and avoiding treatment can result in poorer management of chronic diseases, fewer regular checkups, and missed or delayed start of therapy thus deteriorating health conditions. It can also lead to increased complications and poor prognosis. These factors in turn can increase future health care needs in the region. The strain on local health care resources due to the COVID-19 pandemic is a serious challenge, and treatment interruption may be an indirect burden on health care resources due to COVID-19. Thus, reducing treatment interruption for manageable diseases may alleviate downstream consequences on the health care system.

The findings of this study indicate that controlling the level of infection in a community has important implications for treatment interruption. With the COVID-19 pandemic expected to continue for some time, sustained control of community-level spread will protect populations from the indirect effects of COVID-19, which include treatment interruption. In addition, strategies are needed to prevent treatment interruption. For example, telemedicine has and will continue to play a major role in the provision of health care during the COVID-19 pandemic (22–25). Furthermore, educating patients to avoid treatment interruption and widespread use of long-term prescriptions to prevent patients from running out of regular medications may help avoid health care problems caused by treatment interruption.

A major strength of this study was the relatively large sample size, which allowed us to show, for the first time, an association between community infection level and treatment interruption.

However, this study also had several limitations. First, because this study conducted a cross-sectional study, causality could not be determined. However, since it is theoretically unlikely that treatment interruption experienced by an individual will increase the COVID-19 infection rate in a region, we think it is likely that high regional infection rates cause treatment interruption. Second, the results of this study may not be representative of those of Japan as a whole because this study did not use random sampling. Third, this study did not identify workers' reasons for discontinuing treatment in this study. As discussed above, there are various possible causes of treatment interruption, which may vary by region. Finally, this study did not inquire about the diseases being treated. Treatment interruption may vary depending on the presence or absence of symptoms and the potential disadvantages of discontinuing treatment for a particular disease.

## Conclusions

The present study found that higher regional infection levels were linked to more workers experiencing treatment interruption during the third wave of COVID-19 infection in Japan. Although Further study is needed to clarify the relationship between the kinds of chronic disease, the degree of disease, and treatment interruption, as well as the causes of such interruption, our findings suggest that in addition to individual factors such as socioeconomic status and health status, high regional infection levels may contribute to behavioral changes in the local population, leading to treatment interruption. Preventing community spread of COVID-19 may thus be useful for avoiding treatment interruption for chronic diseases, an emerging medical problem brought about by COVID-19.

## Data availability statement

The raw data supporting the conclusions of this article will be made available by the authors, without undue reservation.

## Ethics statement

The studies involving human participants were reviewed and approved by the Ethics Committee of the University of Occupational and Environmental Health, Japan (Reference Nos. R2-079 and R3-006). Written informed consent for participation was not required for this study in accordance with the national legislation and the institutional requirements.

## Author contributions

JA: conceptualization and writing—original draft. AH, ST, and TN: funding acquisition, project administration, and writing—review and editing. MT, AO, and SM: funding acquisition, project administration, and supervision. MK: supervision and writing—review and editing. YF: conceptualization, data curation, formal analysis, funding acquisition, investigation, methodology, project administration, supervision, writing—original draft, and writing—review and editing. All authors read and approved the final manuscript.

## Funding

This study was supported and partly funded by the research grant from the University of Occupational and Environmental Health, Japan; Japanese Ministry of Health, Labor and Welfare (H30-josei-ippan-002, H30-roudou-ippan-007, 19JA1004, 20JA1006, 210301-1, and 20HB1004); Anshin Zaidan, the Collabo-Health Study Group, and Hitachi Systems, Ltd., and scholarship donations from Chugai Pharmaceutical Co., Ltd. The funder was not involved in the study design, collection, analysis, interpretation of data, the writing of this article or the decision to submit it for publication.

## Conflict of interest

The authors declare that the research was conducted in the absence of any commercial or financial relationships that could be construed as a potential conflict of interest.

## Publisher's note

All claims expressed in this article are solely those of the authors and do not necessarily represent those of their affiliated organizations, or those of the publisher, the editors and the reviewers. Any product that may be evaluated in this article, or claim that may be made by its manufacturer, is not guaranteed or endorsed by the publisher.
